# Hyperparathyroidism-Jaw Tumor Syndrome: A Rare Case

**DOI:** 10.7759/cureus.105352

**Published:** 2026-03-16

**Authors:** Kunal Gupta, Adlyne Reena Asirvatham, Shriraam Mahadevan, Sandhya Sundaram, Dorai Kumar, Narayanan Cunnigaiper

**Affiliations:** 1 Endocrinology, Diabetes and Metabolism, Sri Ramachandra Institute of Higher Education and Research, Chennai, IND; 2 Endocrinology, Sri Ramachandra Institute of Higher Education and Research, Chennai, IND; 3 Pathology, Sri Ramachandra Institute of Higher Education and Research, Chennai, IND; 4 Orthopedic Surgery, Sri Ramachandra Institute of Higher Education and Research, Chennai, IND; 5 General Surgery, Sri Ramachandra Institute of Higher Education and Research, Chennai, IND

**Keywords:** cdc73, fracture, hypercalcemia, hyperparathyroidism, jaw tumor

## Abstract

Hyperparathyroidism-jaw tumor (HPT-JT) syndrome is an uncommon autosomal dominant disorder associated with primary hyperparathyroidism due to parathyroid adenomas and ossifying jaw tumors. We present the case of a 31-year-old male who was incidentally diagnosed with severe parathyroid hormone-dependent hypercalcemia following a traumatic upper limb fracture. Subsequent evaluation confirmed the diagnosis of HPT-JT syndrome. Early recognition, prompt surgical management, and genetic confirmation are essential to ensure appropriate treatment and prevent long-term complications.

## Introduction

Primary hyperparathyroidism (PHPT) is a common endocrine disorder characterized by excessive secretion of parathyroid hormone (PTH), leading to hypercalcemia and its systemic manifestations. In the majority of cases, PHPT results from a solitary benign adenoma (80-85%), with less frequent etiologies including multiglandular hyperplasia or parathyroid carcinoma [1,2]. However, PHPT may also occur as a component of hereditary or syndromic disorders, such as multiple endocrine neoplasia type 1 (MEN1), multiple endocrine neoplasia type 2A (MEN2A), or hyperparathyroidism-jaw tumor (HPT-JT) syndrome [3,4].

HPT-JT syndrome is a rare autosomal dominant disorder associated with germline mutations in the CDC73 (previously known as HRPT2) gene, which encodes the tumor suppressor protein parafibromin [5]. Loss of function of parafibromin leads to dysregulation of transcriptional control and cellular proliferation, predisposing affected individuals to parathyroid tumors (including adenomas and carcinomas) and ossifying fibromas of the jaw. Additional manifestations may include renal cysts or neoplasms, as well as uterine tumors in females [5,6]. The lifetime risk of parathyroid carcinoma in CDC73 mutation carriers has been estimated to be between 15% and 20%, which is substantially higher than in other forms of PHPT [5,7].

Clinically, HPT-JT often presents with asymptomatic hypercalcemia, incidentally detected in up to 80% of individuals [8]. When symptomatic, patients exhibit classic features of hypercalcemia described as “bones, stones, abdominal groans, and psychic moans,” encompassing skeletal pain or fractures, nephrolithiasis, gastrointestinal complaints, and neuropsychiatric disturbances [1,2]. Nephrolithiasis and bone demineralization primarily result from sustained elevations in PTH, whereas anorexia, nausea, constipation, and polyuria are direct consequences of hypercalcemia [2].

Long-term population-based studies have demonstrated that PHPT is associated with an increased risk of fractures and decreased bone mineral density, particularly in cortical bone sites such as the forearm and hip [9]. A Swedish cohort study spanning 19 years revealed a significantly higher incidence of fractures among men with PHPT, emphasizing the sex-based differences in skeletal outcomes [9]. Despite its rarity, HPT-JT holds critical diagnostic and prognostic importance because it may masquerade as sporadic PHPT while carrying a high malignant potential. Therefore, identification of CDC73 mutations in young patients or those with recurrent, multiglandular, or atypical parathyroid lesions is essential for appropriate surveillance, genetic counseling, and familial screening [3-6]. This case highlights the importance of recognizing syndromic causes of hyperparathyroidism, particularly in young male patients, to enable early detection and intervention.

## Case presentation

A 31-year-old male, married with two children, arrived at the emergency department following a road traffic accident. He suffered a fracture of the right shaft of the humerus. His wife, who was a passenger during the motorcycling accident, sustained no major injuries. He had no prior history of fractures or known metabolic bone disease. His surgical history includes a right hemimandibulectomy conducted 12 years prior, with histological verification of a cemento-ossifying fibroma of the mandible.

The patient reported no history of renal or gallbladder stones, pancreatitis, or any systemic diseases. There was no family history of similar complaints. On clinical examination, a hard nodule was palpable in the right neck; the rest of the systemic examination was unremarkable.

A diagnosis of PTH-dependent hypercalcemia was confirmed using Roche's second-generation PTH assay. Ultrasonography of the neck identified two nodules: one located inferior to the right inferior thyroid gland and another positioned next to the left inferior thyroid. Subsequent localization with technetium-99m sestamibi scintigraphy revealed significant absorption in the left inferior parathyroid area, indicative of an adenoma.

The coexistence of biochemical hyperparathyroidism and a previous ossifying fibroma raised significant suspicion for HPT-JT syndrome. The patient underwent surgical exploration. During surgery, two separate adenomas were located and removed from the left and right inferior parathyroid glands. Following excision of the second adenoma, intraoperative PTH levels decreased from 2705 pg/mL to 117 pg/mL, a reduction exceeding 50%, indicative of effective resection. Histopathological examination confirmed the diagnosis of parathyroid adenoma in both glands. Postoperatively, the patient experienced hungry bone syndrome, marked by symptomatic hypocalcemia and hypomagnesemia, necessitating calcium and magnesium supplementation. Genetic testing identified a heterozygous mutation in exon 15 of the CDC73 gene, which is likely pathogenic for the given condition, thereby confirming the diagnosis of familial HPT-JT syndrome. Laboratory investigations (Table 1) confirmed the diagnosis of PTH-dependent hypercalcemia.

**Table 1:** Biochemical investigations

Laboratory marker	Value	Normal reference range
Serum calcium	14.0 mg/dL	8.5-10.5 mg/dL
Serum phosphate	2.3 mg/dL	3.5-5.5 mg/dL
Parathyroid hormone	2705 pg/mL	15-75 pg/mL
Vitamin D	16 ng/mL	20-50 ng/mL
Serum creatinine	1.4 mg/dL	0.6-1.2 mg/dL
Alkaline phosphatase	1400 U/L	30-120 U/L
Serum prolactin	20 ng/mL	4-15 ng/mL (male)

**Figure 1:**
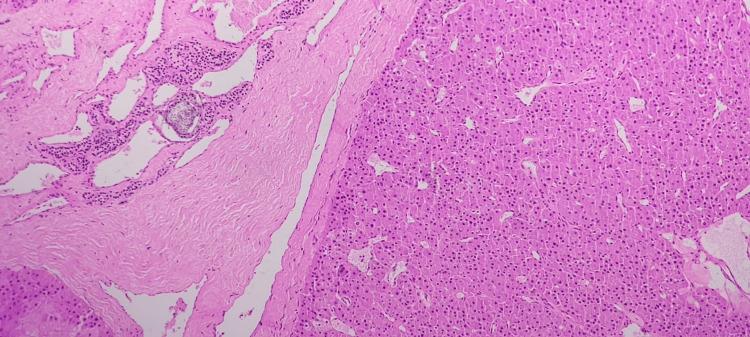
Sections from the left parathyroid gland show a parathyroid adenoma with a well-defined capsule and adjacent compressed parathyroid tissue, as seen on H&E stain at x200. H&E: hematoxylin and eosin

**Figure 2:**
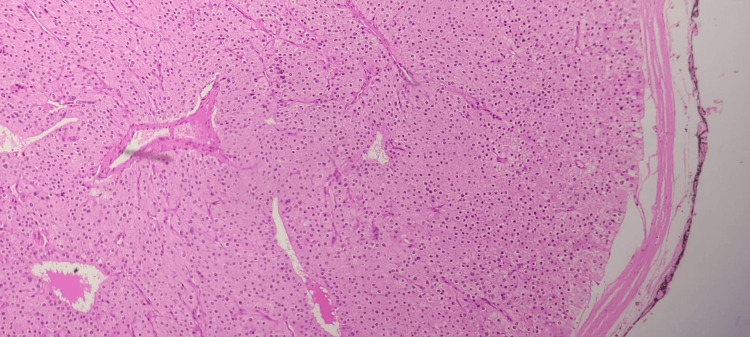
Sections from the right parathyroid gland also show a well-encapsulated parathyroid adenoma, characterized by cells with abundant cytoplasm and a round to oval nucleus, as seen on H&E stain at x200. H&E: hematoxylin and eosin

Histopathological examination (Figures 1-2) confirmed the presence of a parathyroid adenoma in both glands.

## Discussion

The parathyroid glands are responsible for the production of PTH, which plays a role in regulating calcium and phosphate levels in the body. The majority of its actions occur at the bone, where it stimulates bone resorption, which in turn leads to the release of calcium and phosphate. Additionally, it facilitates calcium resorption in the kidneys, specifically in the distal tubule, and induces phosphaturia [8]. An increase in calcium levels, accompanied by a decrease in phosphate levels, is observed in PHPT. PHPT is characterized by a high plasma concentration of intact PTH in conjunction with hypercalcemia [9].

HPT-JT is an autosomal dominant condition characterized by incomplete penetrance. Patients exhibit parathyroid tumors, ossifying fibromas of the jaw, and various renal and uterine tumors [10,11]. This is attributed to a mutation in the CDC73 tumor suppressor gene, which encodes the protein parafibromin, responsible for cell cycle arrest [10]. This is an exceptionally rare syndrome, with approximately 100 documented cases of CDC73 mutation carriers to date [12]. Patients with HPT-JT exhibit parathyroid cancer or adenoma at a mean onset age of 33 years and have a higher risk of parathyroid carcinoma [10]. In this instance, the patient suffered a pathological fracture because of severe PHPT. Additionally, the patient had a history of ossifying fibroma, which further suggests the possibility of a syndromic illness. The radiographic findings of parathyroid adenomas, in conjunction with the considerable increase in serum calcium and PTH levels, suggested that the patient was suffering from a serious and ongoing disease.

In patients who have PHPT, vitamin D deficiency is significantly more prevalent. This can be attributed to several reasons, including an increased conversion of 25-hydroxy vitamin D to 1,25-dihydroxy vitamin D and increased hepatic inactivation of 25-hydroxy vitamin D levels [13,14]. Therefore, people who are suffering from a significant vitamin D deficiency at the same time often have more pronounced biochemical and clinical signs of PHPT. The histological confirmation is essential, as parathyroid carcinomas have been described with increasing frequency in HPT-JT patients.

According to our observations, double adenomas are not uncommon in HPT-JT and require a full intraoperative assessment. This was the situation in this particular case. To confirm biochemical remission following excision, intraoperative PTH monitoring is essential. The postoperative appearance of hungry bone syndrome, which is a known outcome following parathyroidectomy in cases of persistent and severe hyperparathyroidism, necessitated careful monitoring and the administration of replacement therapy. Previous literature indicates that in instances of severe or symptomatic hypercalcemia, intravenous fluids and bisphosphonate infusions have been employed as supportive therapies [15]. Furthermore, the calcimimetic drug cinacalcet hydrochloride has been utilized in the treatment of carcinomas [16,17]. Surgical resection remains the primary treatment for ossifying fibromas of the jaw, with the surgical extent customized according to the tumor's size, location, and related symptoms [18]. Renal and uterine symptoms have been addressed in accordance with established therapeutic protocols, which frequently incorporate routine surveillance and surgical intervention as needed.

Based on the available literature, it has been shown that the majority of CDC73 mutations comprise frameshift, nonsense, and missense variants, as well as small deletions and changes [19]. On the other hand, our patient had a full gene deletion that encompassed exons 1-17 of the CDC73 gene, a situation rarely reported in the scientific literature [20]. Given that gross deletions are only described in one percent of CDC73 mutation cases, this specific incidence is particularly notable [20].

## Conclusions

HPT-JT syndrome is a rare but clinically significant hereditary disorder with important diagnostic, therapeutic, and prognostic implications. A high index of suspicion is warranted in younger individuals presenting with PHPT, particularly those with a history of jaw tumors or a positive family history suggestive of syndromic disease. A comprehensive evaluation, including detailed clinical history, biochemical assessment, and imaging, is crucial for timely identification. Early surgical intervention guided by intraoperative PTH monitoring remains the cornerstone of management, aiming to prevent recurrence and malignant transformation.

Genetic confirmation through CDC73 mutation analysis not only establishes the diagnosis but also facilitates family screening, genetic counseling, and long-term surveillance for associated renal and uterine manifestations. This case underscores the importance of multidisciplinary collaboration among endocrinologists, surgeons, pathologists, and geneticists in achieving optimal outcomes. Increased clinician awareness and routine consideration of HPT-JT in atypical or severe presentations of hyperparathyroidism can lead to earlier detection, better management, and improved prognostic outcomes for affected individuals.
